# Organotrialkoxysilane-Functionalized Noble Metal Monometallic, Bimetallic, and Trimetallic Nanoparticle Mediated Non-Enzymatic Sensing of Glucose by Resonance Rayleigh Scattering

**DOI:** 10.3390/bios11040122

**Published:** 2021-04-15

**Authors:** Prem C. Pandey, Murli Dhar Mitra, Shubhangi Shukla, Roger J Narayan

**Affiliations:** 1 Department of Chemistry, Indian Institute of Technology (BHU), Varanasi 221005, India; murlidharmitra.rs.chy17@itbhu.ac.in (M.D.M.); shubhangi.rs.chy14@itbhu.ac.in (S.S.); 2 Joint Department of Biomedical Engineering, University of North Carolina, Chapel Hill, NC 27599-7575, USA

**Keywords:** organotrialkoxysilane, bimetallic and trimetallic nanoparticles, resonance Rayleigh scattering, synchronous fluorescence spectroscopy, glucose sensing

## Abstract

Organotrialkoxysilanes like 3-aminopropyltrimethoxysilane (3-APTMS)-treated noble metal cations were rapidly converted into their respective nanoparticles in the presence of 3-glycidoxypropylytrimethoxysilane (3-GPTMS). The micellar activity of 3-APTMS also allowed us to replace 3-GPTMS with other suitable organic reagents (e.g., formaldehyde); this approach has significant advantages for preparing bimetallic and trimetallic analogs of noble metal nanoparticles that display efficient activity in many practical applications. The formation of monometallic gold, silver, and palladium nanoparticles, bimetallic Ag-Pd, and Au-Pd nanoparticles at various ratios of noble metal cations, and trimetallic Ag-Au-Pd nanoparticles were studied; their biocatalytic activity in non-enzymatic sensing of glucose based on monitoring synchronous fluorescence spectroscopy (SFS) was assessed. Of these nanoparticles, Au-Pd made with an 80:20 Au:Pd ratio displayed excellent catalytic activity for glucose sensing. These nanoparticles could also be homogenized with Nafion to enhance the resonance Rayleigh scattering (RRS) signal. In this study, the structural characterization of noble metal nanoparticles as well as bi- and tri-metallic nanoparticles in addition to their use in non-enzymatic sensing of glucose are reported.

## 1. Introduction

Noble metal nanoparticles with surface functionalization by a organotrialkoxysilane (e.g., 3-aminopropyltrimethoxysilane (3-APTMS) and 3-glycidoxypropylytrimethox ysilane (3-GPTMS)) have potential use in catalytic applications [1,2,3,4,5,6,7,8,9,10,11,12,13,14,15]. The use of 3-APTMS and 3-GPTMS to synthesize noble metal nanoparticles has previously been demonstrated [1,2,3,4,5,6,7,8,9,10,11,12,13]. It has been reported that 3-APTMS capped gold ions are converted into nanoparticles in the presence of reducing agents such as 3-GPTMS, cyclohexanone, tetrahydrofuran hydroperoxide, and formaldehyde. The controlled conversion of gold cations to gold nanoparticles within one minute was enabled by the reducing functionality of 3-APTMS, 3-GPTMS, cyclohexanone, and formaldehyde. We demonstrated that 3-APTMS in the presence of cyclohexanone allows for the conversion of potassium ferricyanide to Prussian blue; these studies showed the reducing capability of cyclohexanone in the presence of 3-APTMS [14,15] to convert Fe^+3^ to Fe^+2^. Uppal et al. found that cyclohexanone enables gold cations to be converted into nanoparticles [16]. We have demonstrated that AuNPs that were prepared with cyclohexanone undergo rapid agglomeration, which can be controlled by the presence of 3-APTMS; this method allow gold or palladium cations to be converted into nanoparticles [15]. The micellar behavior of 3-APTMS enables the conversion of hydrophilic cations to stable AuNPs in the presence of hydrophobic cyclohexanone; this method is appropriate for many applications [5,13]. These studies indicated that organotrialkoxysilanes can function as reducing and stabilizing agents for the controlled conversion of noble metal cations into nanoparticles [13]. A similar process efficiently enables the controlled synthesis of other noble metal nanoparticles such as silver nanoparticles and palladium nanoparticles [1,2,3,4,5,6,7,8,9,10,11,12,13]. Synergistic interactions associated with bimetallic and trimetallic nanoparticles were shown to be associated with dramatic changes in catalytic performances [7,8,9,10]. The organotrialkoxysilane reducing agent can be used for the processing of monometallic, bimetallic, [13], and trimetallic nanoparticles [10,13]. Organotrialkoxysilane functionalized bimetallic Ag-Au, Au-Ag, Au-Pd, and Pd-Au nanoparticles were shown to exhibit catalytic ability [11,13] for many applications.

It has been shown that gold nanoparticles can be prepared using a wide range of organotrialkoxysilane concentrations; this approach may be used to control the Resonance Rayleigh scattering (RRS) intensity and enable enzyme-free catalysis of the analyte [17,18,19,20]. This method is straightforward, stable, and selective; it offers an excellent linear dynamic range under physiological conditions, particularly for glucose sensing. The synchronous fluorescence signal recorded from organotrialkoxysilane-functionalized gold nanoparticles was found linearly dependent on the concentration of glucose. Accordingly, a study was undertaken to understand organotrialkoxysilane-functionalized monometallic, bimetallic, and tri-metallic noble metal nanoparticle-mediated sensing of glucose by synchronous fluorescence spectroscopy (SFS). These novel findings are described for the first time in this study.

## 2. Materials and Methods

### 2.1. Materials

Formaldehyde, ethylene glycol, polyvinylpyrrolidone (PVP), methanol, 3-glycidoxyporpyltrimethoxysilane (3-GPTMS), sodium borohydride (NaBH_4_)_,_ 3-aminopropyletrimethoxysilane (3-APTMS), and trachloropalladate (K_2_PdCl_4_) were purchased from Sigma Aldrich Chemicals Pvt Ltd. (Bangalore, India). Methanol, silver nitrate, and tetrachloroauric acid were purchased from Himedia Laboratories (Mumbai, India). Phosphate buffer solution and double-distilled water were used in the experiments.

### 2.2. Synthesis of Organofunctionalized Noble Metal Nanoparticles and Their Multimetallic Analogues

#### 2.2.1. Gold Nanoparticle Formation Mediated by 3-APTMS and 3-GPTMS

Gold nanoparticles were obtained as mentioned in a previous study [13]. The 3-APTMS capped gold cations in the presence of 3-GPTMS were processed for 10 s in a microwave oven. This step was repeated 1–4 times in order to create a dark red colored gold nanoparticles.

#### 2.2.2. Silver Nanoparticle Formation Mediated by 3-APTMS and 3-GPTMS

Silver nanoparticles were obtained as mentioned in a previous study [13]. The 3-ATPMS capped silver cations in the presence of 3-GPTMS were processed for 20 s in a microwave oven. This step was repeated 3–5 times in order to create a dark yellow colored silver nanoparticle colloidal suspension.

#### 2.2.3. Palladium Nanoparticle Formation Mediated by 3-APTMS and Formaldehyde

Palladium nanoparticles were obtained as mentioned in a previous study [13]. The 3-ATPMS capped palladium cations in the presence of formaldehyde were processed over 8 s in a microwave oven. This step was repeated 3–7 times in order to create a dark black colored palladium nanoparticle colloidal suspension.

#### 2.2.4. Bimetallic Silver–Gold Nanoparticles Mediated by 3-APTMS and 3-GPTMS

Au-Ag bimetallic nanoparticles were obtained as mentioned in a previous study [13]. The gold nanoparticles were made with organotrialkoxysilane, followed by the addition of silver cations as mentioned earlier [13].The resulting mixture was incubated over 10 s in a microwave oven. This step was repeated 3–7 times in order to create a dark yellowish-orange colored bimetallic (Au@Ag) colloidal suspension.

#### 2.2.5. Trimetallic Au-Ag-Pd Nanoparticle Formation Mediated by 3-APTMS, 3-GPTMS, and Formaldehyde

Au-Ag-Pd trimetallic nanoparticles were obtained as mentioned in a previous study [13]. The mixture of bimetallic (Au-Ag) and cations of palladium was mixed under stirring. The mixture was incubated for over 20 s in a microwave oven. This step was repeated 2–7 times in order to create a dark yellowish-orange colored trimetallic Au-Ag-Pd colloidal suspension.

#### 2.2.6. Bimetallic Au-Pd Nanoparticle Formation Mediated by 3-APTMS, 3-GPTMS, and Formaldehyde

200 μL K_2_PdCl4 (0.025 M in ethylene glycol) was mixed under stirring. 200 μL of previously-synthesized gold nanoparticle colloidal suspension (made as discussed in Section 2.2.1) was added as mentioned earlier [13]. A 150 μL measure of formaldehyde was added to the stirring mixture. The mixture was incubated for 6 s in a microwave oven. This step was repeated 4–10 times in order to create a dark yellowish-orange colored colloidal suspension of bimetallic Au-Pd nanoparticles. The synthesis of gold and palladium bimetallic nanoparticles with Au:Pd ratios of 20:80 and 80:20 was performed using a similar procedure.

#### 2.2.7. Bimetallic Ag-Pd nanoparticle Formation Mediated by 3-APTMS, 3-GPTMS, and Formaldehyde

A 200 μL measure of K_2_PdCl_4_ (0.025 M in ethylene glycol) was mixed under stirring conditions; 200 μL of previously-synthesized silver nanoparticle colloidal suspension (made as discussed in Section 2.2.2) was added as described earlier [13]. A 180 μL measure of formaldehyde was added to the stirring mixture. This mixture was incubated for 6 s in a microwave oven. This step was repeated 5–12 times in order to create a dark yellowish-orange colored colloidal suspension of bimetallic Ag-Pd nanoparticles.

#### 2.2.8. Materials Characterization and Spectroscopic Analysis

X-ray diffraction (XRD) data were collected with a Miniflex II diffractometer (Rigaku, Tokyo, Japan). Transmission electron microscopy (TEM) was performed using a JEM-2100F electron microscope (JEOL, Tokyo, Japan). An F7000 fluorescence spectrophotometer (Hitachi, Tokyo, Japan) was used to obtain SFS data. The wavelength interval was maintained at 0 nm (Δλ = 0 nm) to obtain the resonance Rayleigh scattering spectra.

## 3. Results

### 3.1. Organotrialkoxysilane Mediated Synthesis of AuNP, AgNP, and PdNP and Their Multimetallic Analogues

**Figure 1:**
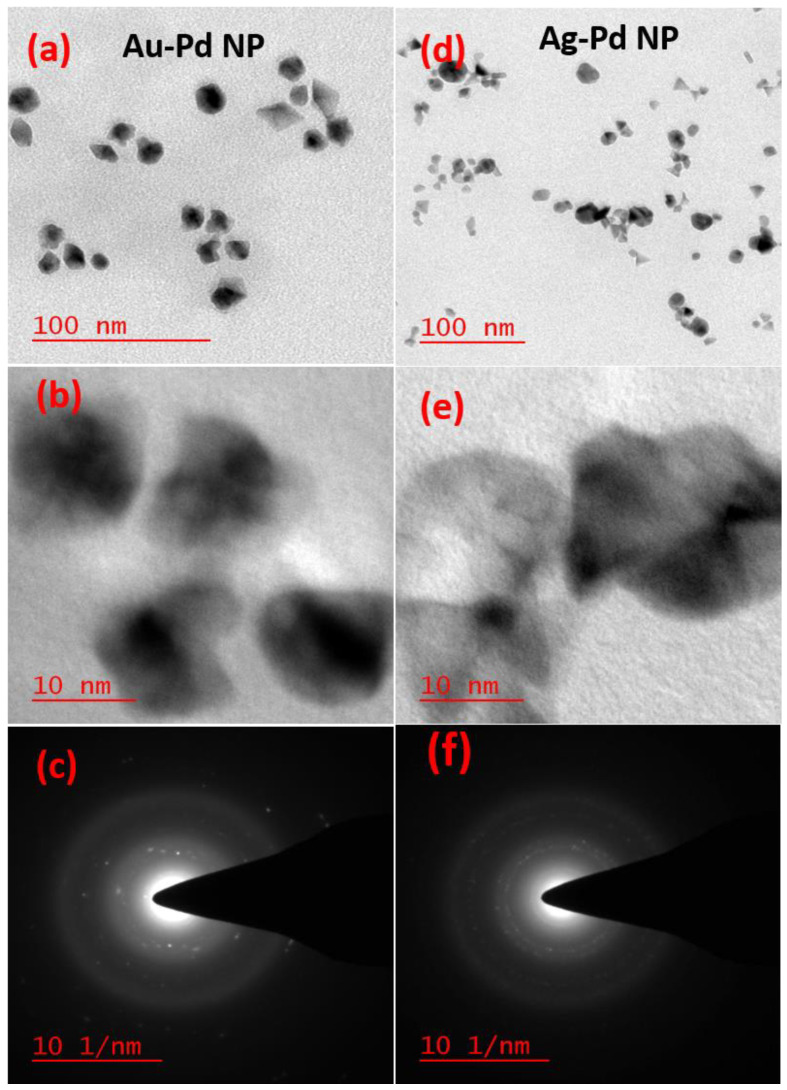
TEM images (**a**,**b**) and corresponding selected area electron diffraction patter (SAED) patterns of bimetallic (Au-Pd) nanoparticles (NPs) (**c**). TEM images (**d**,**e**) and corresponding SAED patterns of bimetallic (Au-Pd) NPs (**f**).

**Figure 2:**
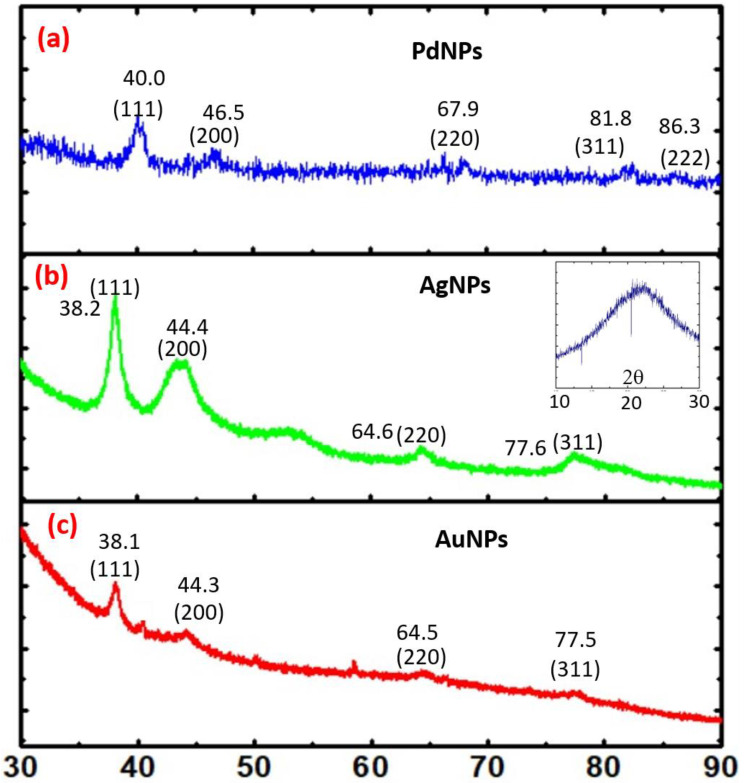
XRD profile of (**a**) PdNPs, (**b**) AgNPs, (**c**) AuNPs.

3-APTMS capped noble metal cations may be converted into monometallic, bimetallic, and trimetallic nanoparticles in the presence of a small organic reducing agent (e.g., cyclohexanone, tetrahydrofuran hydroperoxide, or formaldehyde) or 3-GPTMS [1,2,3,4,5,6,7,8,9,10,11,12,13]. These as-made nanoparticles can be made insensitive to pH changes [10]. Since the functional groups bound to the alkoxysilanes are microwave active, nanoparticles may be processed under microwave incubation; previous studies have demonstrated rapid conversion of metal cations into nanoparticles using this approach [13]. It is noteworthy that microwave processing enables the rapid synthesis of bimetallic nanoparticles and trimetallic nanoparticles with sufficient stability for practical applications; these nanoparticles are shown in the supporting information (Figures S1–S3). Figure 1a,b contain the TEM images of bimetallic (Au-Pd) nanoparticles made at a 80:20 ratio of Au/Pd at two different magnifications; Figure 1c shows the selected area electron diffraction pattern (SAED) patterns from these materials. The TEM images of Ag-Pd bimetallic nanoparticles shown in Figure 1d,e at two different magnifications; the SAED pattern from this material is shown in Figure 1f. Figures S1–S3 contain the TEM images of the monometallic, bimetallic, and trimetallic nanoparticles that were obtained from the organotrialkoxysilane-mediated conversion of the metal cations. Figure S1a,c show the TEM images of the AuNPs and AgNPs, respectively. The SAED patterns from the AuNPs and AgNPs are provided in Figure S1b,d, respectively. Figure S2a shows the TEM image of the Au-Ag NPs; a higher magnification image of the Au-Ag NPs is shown in Figure S2b. A SAED pattern from the Au-Ag NPs is shown in Figure S2c,d shows a TEM image of the PdNPs; a higher magnification image of the PdNPs is shown in Figure 2e. A SAED pattern from the PdNPs is shown in Figures S2f and S3a shows a TEM image of the Au-Ag-Pd NPs; a higher magnification TEM image of the Au-Ag-Pd NPs is shown in Figure S3b. A SAED pattern from the Au-Ag-Pd NPs is shown in Figure S3c. The mechanism of acid-base adduct mediated reduction of noble metal cations is shown in the supporting information (Figure S4).

**Figure 3:**
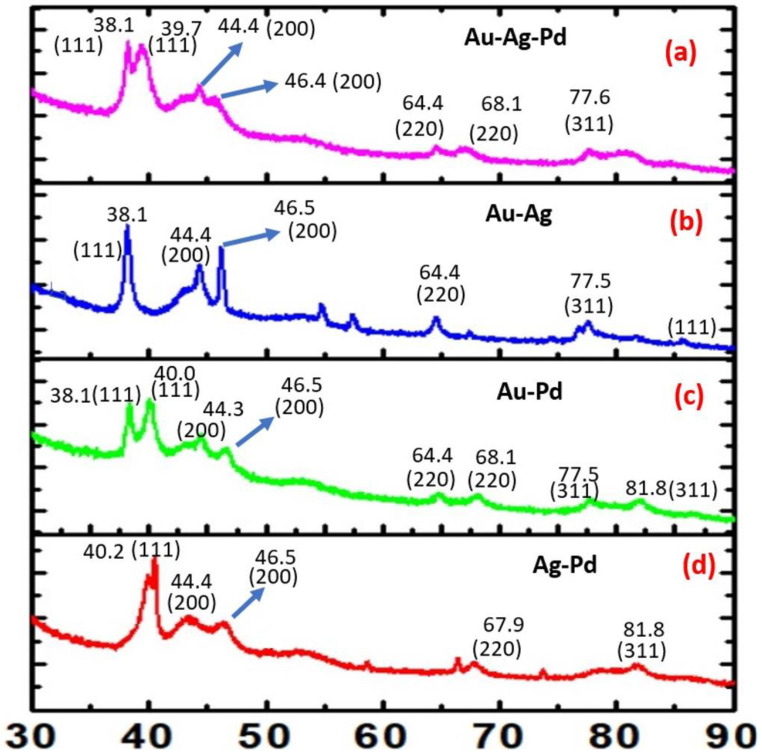
XRD profile of (**a**) trimetallic (Au-Ag-Pd) NPs, (**b**) bimetallic (Au-Ag) NPs, (**c**) bimetallic (Au-Pd) NPs, and (**d**) bimetallic (Ag-Pd) NPs.

These findings confirm that the organotrialkoxysilane efficiently allows the controlled reduction of noble metal cations into nanoparticles in a variety of configurations to yield noble metal nanoparticles and multimetallic nanoparticles. These nanoparticles have been further characterized by XRD analysis. Figure 2 shows the XRD analysis of PdNP (Figure 2a), AgNP (Figure 2b) and AuNP (Figure 2c) nanoparticles, respectively. Similarly, the XRD results from Au-Ag-Pd, Ag-Au, Ag-Pd, Au-Pd nanoparticles are shown in Figure 3a–d, respectively. The corresponding planes as evaluated from diffraction patterns confirm the role of the trialkoxysilane in the controlled processing of noble metal nanoparticles and multimetallic nanoparticles.

### 3.2. Synchronous Fluorescence Spectroscopy of Organotrialkoxysilane-Functionalized Noble Metal Nanoparticles and Multimetallic Nanoparticles for Non-Enzymatic Sensing of Glucose

Synchronous fluorescence spectroscopy has been extensively utilized to detect compounds in solution [14,15,17]; previous studies have demonstrated the detection of blood glucose using gold nanoparticles [16]. These efforts directed us to examine the role of the as-made metal nanoparticles, bimetallic nanoparticles, as well as trimetallic nanoparticles for enzyme-free sensing of glucose [13,15]. Since bimetallic and trimetallic nanoparticles may be using different ratios of noble metal cations with the organotrialkoxysilane, we investigated the performance of as made AuNPs, AgNPs, PdNPs, Ag-Au NPs, Ag-Pd NPs, Au-Pd NPs, and Ag-Au-Pd NPs for enzyme-free sensing of glucose based on SFS measurements. The resonance Rayleigh scattering was monitored by measuring synchronous fluorescence spectroscopy at Δλ = 0 nm. We have recently demonstrated the role of AuNP in hydrazine sensing based on monitoring the SFS intensity [19,20]. The sensitivity of the SFS signal increased with a decrease in the size of the AuNPs [19,20]. Accordingly, modulation of the physical properties of the AuNPs may be used to alter the sensitivity of the SFS signal. Efforts are underway to modulate the physical properties of the AuNPs in order to increase the SFS response as a function of analyte concentration; detailed finding related to this effort will be communicated in next submission.

**Figure 4:**
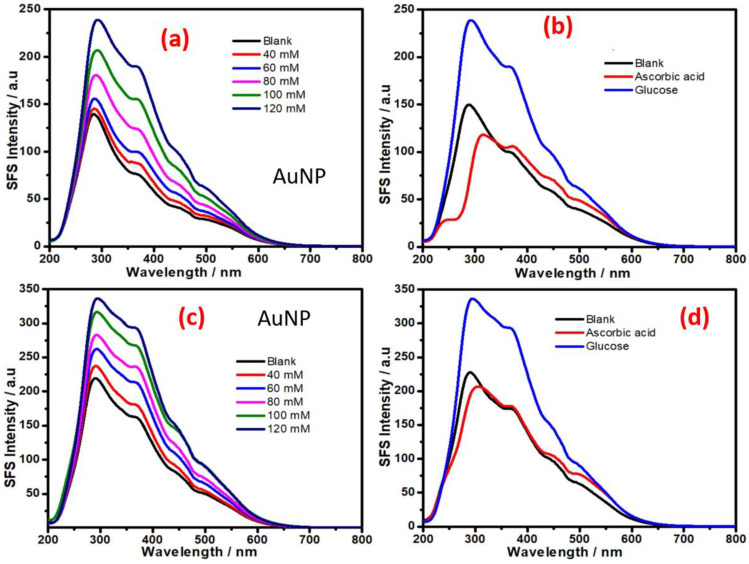
(**a**) Synchronous fluorescence spectra at delta δλ = 0 nm; the spectra were recorded at various concentrations of glucose. (**b**) Synchronous fluorescence intensity of AuNPs in the presence of glucose and ascorbic acid. (**c**) Synchronous fluorescence spectra at delta δλ = 0 nm; the spectra were recorded at different concentrations of glucose with Nafion. (**d**) Synchronous fluorescence intensity of AuNPs with Nafion in the presence of glucose and ascorbic acid.

Figure 4a shows the SFS signal of organotrialkoxysilane-stabilized AuNPs in the absence and the presence of various concentrations of glucose. Figure 4b shows the SFS signal in the presence of glucose and the interferent ascorbic acid; this result shows the selectivity of the approach for non-enzymatic sensing of glucose. The micellar activity of the organotrialkoxysilane allows the incorporation of an ion exchanger such as Nafion with the organotrialkoxysilane-stabilized gold nanoparticles. Figure 4c,d show the results from the Nafion incorporated gold nanoparticles; these results indicate the dependence of the SFS signal on the glucose concentration. Additionally, the results show an enhancement in the SFS signal for glucose in the presence of Nafion; moreover, the SFS signal for ascorbic acid (5 mM) is significantly reduced. These results show the effectiveness of this AuNP for enzyme-free sensing of glucose.

**Figure 5:**
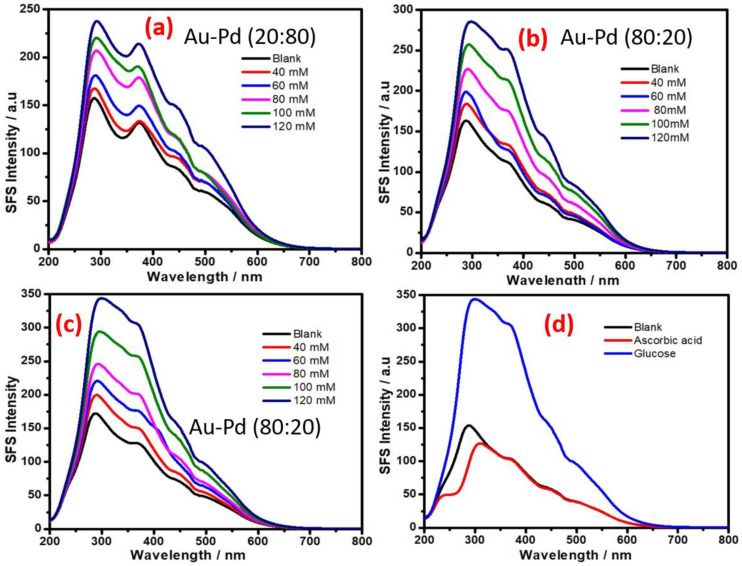
Synchronous fluorescence spectra at delta δλ = 0 nm; the spectra were recorded at various concentrations of glucose. (**a**) Synchronous fluorescence intensity of bimetallic (Au-Pd) NPs with an Au:Pd ratio of 20:80. (**b**) Synchronous fluorescence intensity of bimetallic (Au-Pd) NPs with an Au:Pd ratio of 80:20. (**c**) Synchronous fluorescence intensity of bimetallic (Au-Pd) NPs with an Au:Pd ratio of 80:20 with Nafion. (**d**) Synchronous fluorescence intensity of bimetallic (Au-Pd) NPs with an Au:Pd ratio of 80:20 with Nafion in the presence of glucose and ascorbic acid.

**Figure 6:**
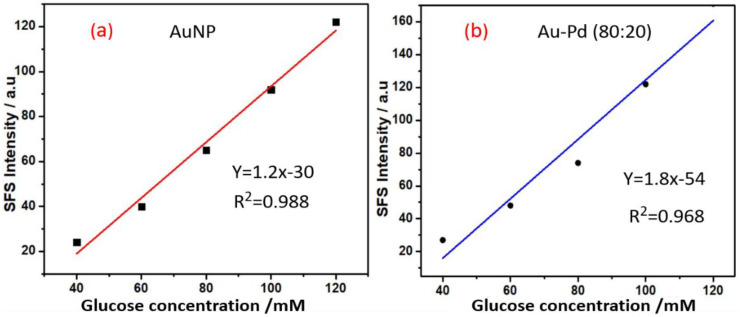
Linear plot (**a**) Concentration of glucose versus synchronous fluorescence spectroscopy (SFS) intensity in the presence of AuNPs with Nafion. (**b**) The concentration of glucose versus SFS intensity in the presence of bimetallic Au-Pd nanoparticles with an Au:Pd ratio of 80:20 with Nafion.

The role of other metal nanoparticles and multimetallic nanoparticles for enzyme-free sensing of glucose was also assessed. Since AuNPs display excellent SFS activity for enzyme-free sensing of glucose (Figure 4), we attempted to understand the role of bimetallic Au-Pd NPs made with different ratios of both metal cations. To understand the variation of the SFS signal with the composition of the bimetallic nanoparticles, Au-Pd bimetallic nanoparticles were made with 20:80 and 80:20 gold:palladium ratios. The results from the variation of the SFS signal as a function of glucose concentration with as-made bimetallic nanoparticles are shown in Figure 5; the result from the as-made bimetallic nanoparticles with an 20:80 Au:Pd ratio is shown in Figure 5a and the result of nanoparticles with the same made at 80:20 Au:Pd ratio is shown in Figure 5b. These results confirm that an increase in the palladium content results in a decrease in the SFS signal; the bimetallic NPs made with an 80:20 Au:Pd ratio show better sensitivity than that made at 20:80 ratio of Au:Pd and also to that of only AuNPs (Figure 4); this result indicates the utility of these bimetallic nanoparticles for glucose sensing. The incorporation of Nafion with the bimetallic nanoparticles made with 80:20 of Au:Pd shows better sensitivity for glucose sensing (Figure 5c) as compared to that in absence of Nafion (Figure 5b). The calibration curves for the analysis of glucose based on SFS intensity (Δλ = 0 nm) using AuNP at wavelength 369 nm is shown in Figure 6a; similar results with Au-Pd bimetallic NPs made with a 80:20 ratio at a wavelength of 371 nm is shown in Figure 6b. these results confirm that the bimetallic Au-Pd nanoparticles have high sensitivity for glucose analysis in presence of Nafion and also justify the red shift for better enzyme free sensing of glucose.

**Figure 7:**
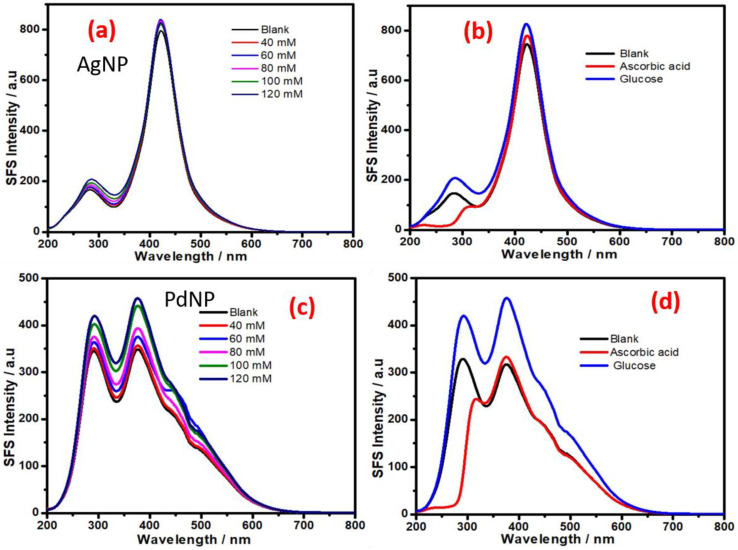
Synchronous fluorescence spectra at delta δλ = 0 nm, recorded at various concentrations of glucose. (**a**) Synchronous fluorescence intensity of AgNPs in the presence of glucose and ascorbic acid. (**b**) Synchronous fluorescence spectra at delta δλ = 0 nm recorded at various concentrations of glucose. (**c**) Synchronous fluorescence intensity of PdNPs in the presence of glucose and ascorbic acid (**d**).

**Figure 8:**
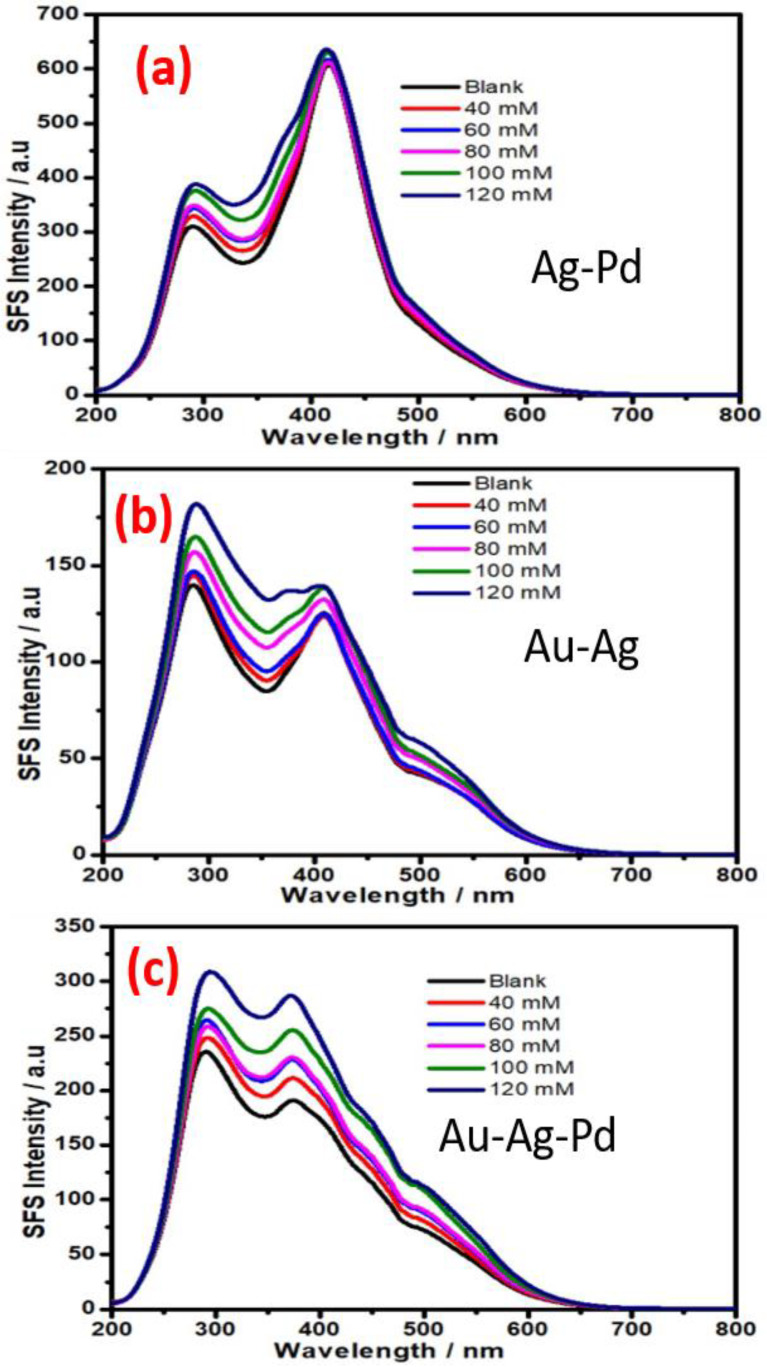
Synchronous fluorescence spectra at δλ = 0 nm recorded at various concentration of glucose: (**a**) bimetallic (Ag-Pd) NPs, (**b**) bimetallic (Ag-Au) NPs, and (**c**) trimetallic (Au-Ag-Ap) NPs.

We also investigated the role of other monometallic, bimetallic and trimetallic nanoparticles (e.g., Ag NPs, Pd NPs, Ag-Au NPs, and Ag-Au-Pd NPs) to understand their SFS signal as a function of the glucose concentration. The results recorded in Figure 7 indicate the dependence of the SFS signal on the glucose concentration with AgNPs (Figure 7a) and PdNPs (Figure 7c), respectively. Additionally, the results in the presence of ascorbic acid have been shown in Figure 7b,d, respectively. These results indicate that AgNPs are the least sensitive in terms of the SFS signal produced as a function of the glucose concentration; PdNPs were noted to show better sensitivity than AgNPs (Figure 7). Furthermore, the presence of gold and silver as Ag-Au bimetallic NPs also significantly reduces the sensitivity of the SFS signal as a function of the glucose concentration (Figure 8); this result suggests the presence of silver was associated with a decrease in SFS signal as a function of the glucose concentration.

The variation of the SFS signal from Ag-Pd, Ag-Au, and Au-Ag-Pd NPs as a function of the glucose concentration is shown in Figure 8. Figure 8a–c indicate that the bimetallic and trimetallic NPs that contain Ag as one of constituents are not potentially active for non-enzymatic glucose sensing based on synchronous fluorescence spectroscopy (Figure 8); this result confirm the finding related to the role AgNP as recorded in Figure 7a.

## 4. Conclusions

This study describes organotrialkoxysilane-mediated synthesis of monometallic, bimetallic, and trimetallic noble metal nanoparticles with high stability for potential use in enzyme free detection of glucose based on synchronous fluorescence spectroscopy (SFS). The impact of the monometallic, bimetallic, and trimetallic noble metal nanoparticles, AuNPs, AgNPs, PdNPs, Ag-Au NPs, Au-Pd NPs, Ag-Pd NPs, and Au-Ag-Pd NPs) on the variation of the SFS signal for non-enzymatic sensing of glucose was demonstrated. The finding predicts that bimetallic Au-Pd NPs made with an 80:20 Au:Pd ratio display excellent results for glucose sensing. The micellar activity of the as-made nanomaterials can be effectively explored for making Nafion-metal nanoparticles colloidal suspensions for non-enzymatic detection of glucose.

## Data Availability

Data supporting reported results can be found in the laboratory of Prof. Prem C Pandey of IIT(BHU).

## Supplementary Materials

The following are available online at https://www.mdpi.com/article/10.3390/bios11040122/s1, Figure S1: TEM images (a,c) of the AuNPs and AgNPs, respectively. Selected area electron diffraction patterns (c,d) of the AuNPs and AgNPs, respectively, Figure S2: TEM images (a–b) and corresponding SAED patterns of the bimetallic (Au-Ag) NPs (c). TEM images (d–e) and corresponding SAED patterns of the PdNPs (f), Figure S3: TEM images (a–b) and corresponding SAED pattern of the trimetallic (Au-Ag-Pd) NPs (c) and Figure S4: Acid-base adduct mediated reduction of noble metal cations.
