# Supplementary material for: Organotrialkoxysilane-Functionalized Noble Metal Monometallic, Bimetallic, and Trimetallic Nanoparticle Mediated Non-Enzymatic Sensing of Glucose by Resonance Rayleigh Scattering
Source: Biosensors (Basel). 2021 Apr 15;11(4):122. doi: 10.3390/bios11040122 (PMC8071229; doi:10.3390/bios11040122)
Supplement: Supplementary file 1 [file biosensors-11-00122-s001.zip › biosensors-1136444-supplementary.pdf]

Organotrialkoxysilane-Functionalized Noble Metal Monometallic, Bimetallic and Trimetallic Nanoparticle Mediated Non-Enzymatic Sensing of Glucose by Resonance Rayleigh Scattering

P C Pandey[1*], Murli Dhar Mitra[2], Shubhangi Shukla[3], and Roger J Narayan[4*]

Supporting Information

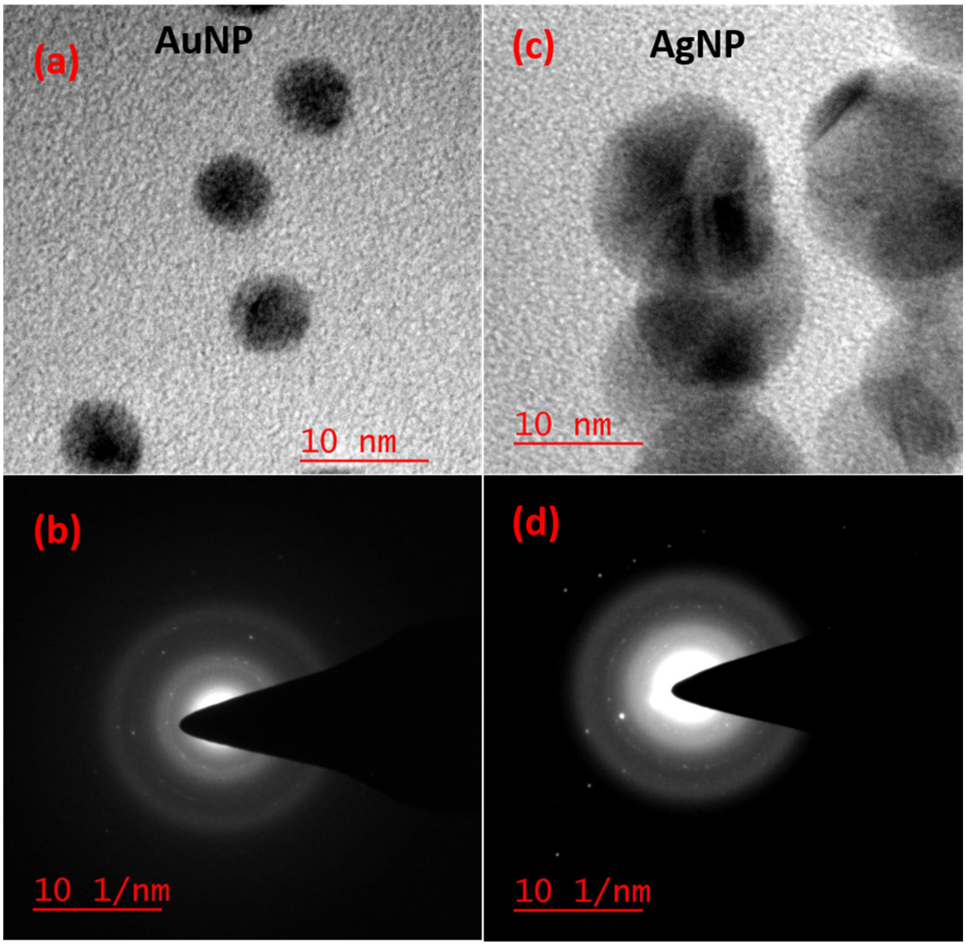

S1. TEM images (a) and (c) of the AuNPs and AgNPs, respectively. Selected area electron diffraction patterns (c) and (d) of the AuNPs and AgNPs, respectively.

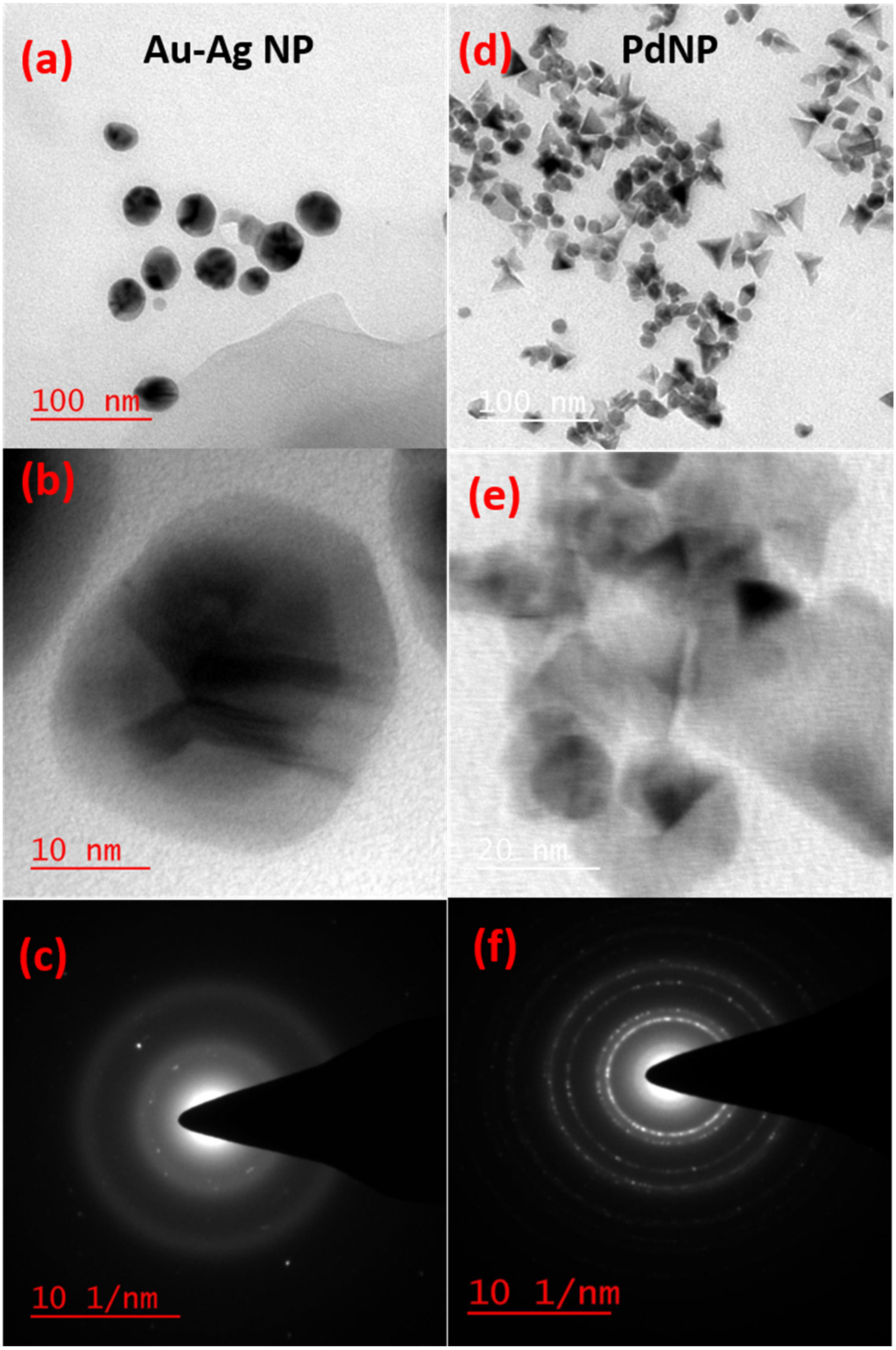

S2. TEM images (a)-(b) and corresponding SAED patterns of the bimetallic (Au-Ag) NPs (c). TEM images (d)-(e) and corresponding SAED patterns of the PdNPs (f).

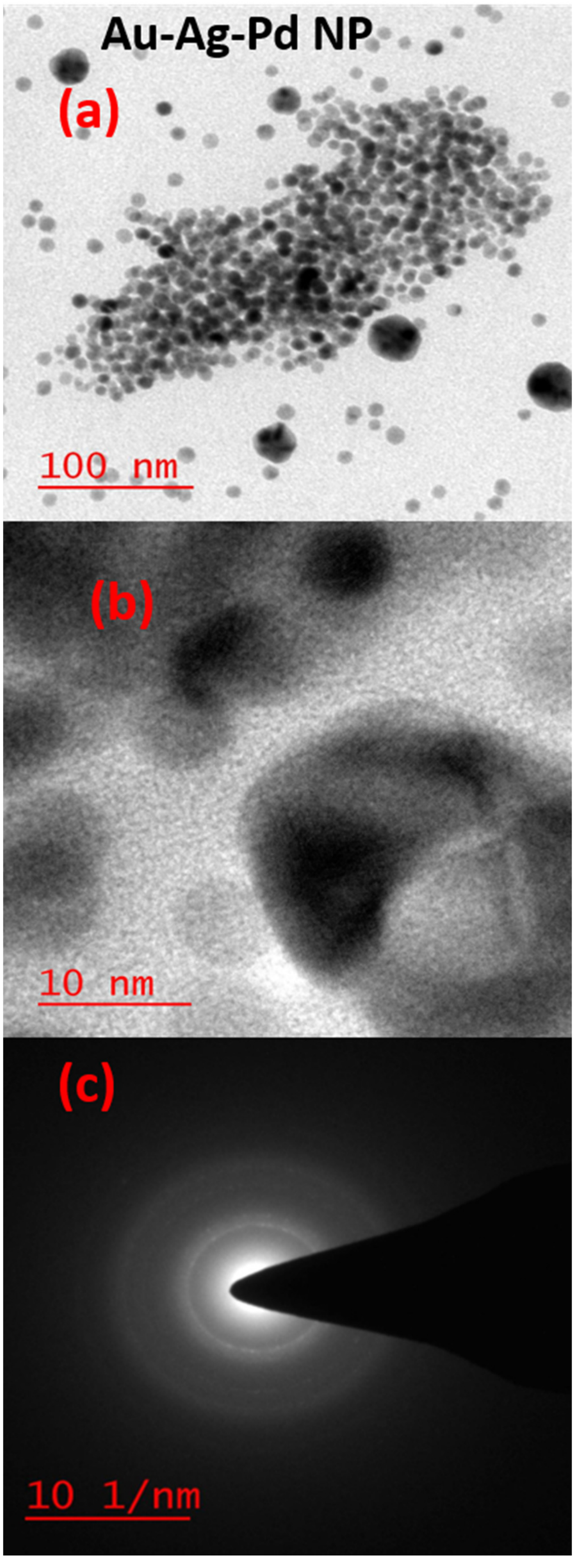

S3. TEM images (a)-(b) and corresponding SAED pattern of the trimetallic (Au-Ag-Pd) NPs (c).

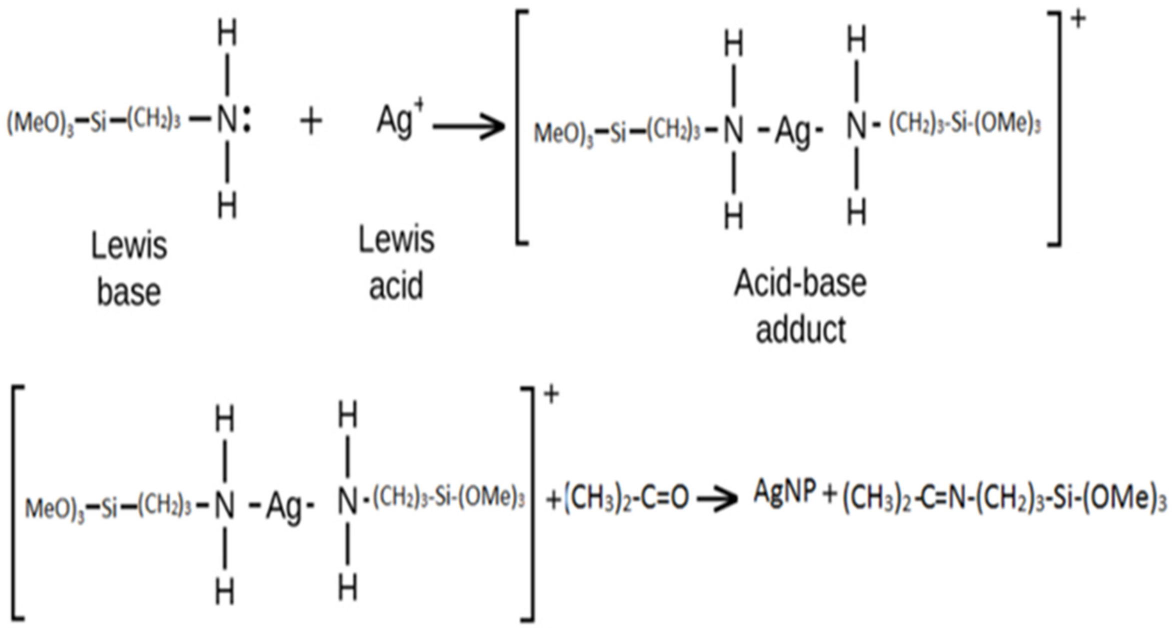

S4. Acid-base adduct mediated reduction of noble metal cations.
